# A Vision of the Possible. What the R.A.M.C. Might Become

**Published:** 1919-12

**Authors:** 


**A Vision of the Possible. What the R.A.M.C. Might Become.** By Sir JAMES W. BARRETT, C.B., M.D., F.R.C.S., late Lieut.-Col., R.A.M.C., etc. Pp. xx., 182. London: H. K. Lewis & Co. 1919. Price 9s. net.

The author served three years in Egypt with the R.A.M.C. as Consulting Aurist and Oculist, and President of a Standing Medical Board, and previously with the Australian Forces, as well as taking an important part in the administration of the Y.M.C.A. and Red Cross.

He discusses various reforms which were carried out in Egypt, which resulted in the saving to the service, then sorely in want of men, of a huge number of perfectly efficient soldiers who were suffering from chronic suppurative otitis, limited vision, or certain orthopædic defects, such as some forms of flat foot without eversion and who would have been lost under the old regulations. He had thousands of the otitis cases treated by mopping out the ear and dropping in daily equal parts of spirit and carbolic lotion (1–15). Hardly any of them developed a general infection in three years. He points out that with reasonable care in selection and treatment the vast majority of these patients do perfectly well. Great fault is found with the earlier medical boards and their waste of good material. One third of the B Class men who arrived in Egypt were at once raised to A category. Ophthalmia, so rife in Egypt, was kept so well under control that in four years only one British soldier lost an eye from it, but when pellagra was present, as in the case of Turkish prisoners and Armenian refugees, it was most disastrous. At one Armenian camp the pellagra was wiped out by raising the calories in the diet. There were very few cases of trachoma, and these nearly all came from Australia. In fact, it was not contracted in Egypt by any British troops. He goes so far as to suggest that the foolishly high standards in unessential things of the English boards were responsible for the shortage of men in France in March, 1918, and the resulting great defeat before Cambrai.

In previous writings he has discussed the management of venereal disease, but here he adds some details of the work done at Port Said, both by prophylaxis and moral means.

There are useful suggestions, too, as to the functions of Red Cross Societies, especially that they should not form or administer hospitals, but confine themselves to providing inquiry bureaus, extra comforts and personal care of the sick, convalescent outings and on on. The thorny question, however, crops up whether they should not in war time cease to be voluntary and amateur bodies, but rather become a part of the R.A.M.C. We seem landed in the old discussion on the comparative merits of voluntary and state hospitals.

As to the organisation of the R.A.M.C., the writer is anxious that it should form in peace a great institution for research and preventive medicine which would attract an able class of men. Promotion both in peace and war should depend on scientific and clinical achievements, as well as on administrative work. It should be possible for a man to rise to the highest rank as a clinician or scientist. At present a great defect is that holders of the highest ranks often cease to be scientific medical men and become merely administrators, out of touch with the progress of their art. The head of a large district should be set free for frequent travelling over it by the appointment of an assistant. Complaint is made, too, of the failure to train new officers, and of the frequent failure to send everywhere with the sick man definite clinical information. An ingenious scheme for promotion is suggested, which he thinks would be attractive and get over many present difficulties.

Some of his proposals seem well worthy of adoption or consideration as meeting recognised defects, but it is fair to add some of the evils have been already removed.

